# Improved sensitivity, accuracy and prediction provided by a high‐performance liquid chromatography screen for the isolation of phytase‐harbouring organisms from environmental samples

**DOI:** 10.1111/1751-7915.13733

**Published:** 2020-12-21

**Authors:** Gregory D. Rix, Jonathan D. Todd, Andrew L. Neal, Charles A. Brearley

**Affiliations:** ^1^ School of Biological Sciences University of East Anglia Norwich Research Park Norfolk NR4 7TJ UK; ^2^ Department of Sustainable Agriculture Science Rothamsted Research Devon EX20 2SB UK

## Abstract

HPLC methods are shown to be of predictive value for classification of phytase activity of aggregate microbial communities and pure cultures. Applied in initial screens, they obviate the problems of ‘false‐positive’ detection arising from impurity of substrate and imprecision of methodologies that rely on phytate‐specific media. In doing so, they simplify selection of candidates for biotechnological applications. Combined with 16S sequencing and simple bioinformatics, they reveal diversity of the histidine phosphatase class of phytases most commonly exploited for biotechnological use. They reveal contribution of multiple inositol‐polyphosphate phosphatase (MINPP) activity to aggregate soil phytase activity, and they identity *Acinetobacter* spp. as harbouring this prevalent soil phytase activity. Previously, among bacteria MINPP was described exclusively as an activity of gut commensals. HPLC methods have also identified, in a facile manner, a known commercially successful histidine (acid) phosphatase enzyme. The methods described afford opportunity for isolation of phytases for biotechnological use from other environments. They reveal the position of attack on phytate by diverse histidine phosphatases, something that other methods lack.

## Graphical abstract

HPLC‐based screening of soil identifies Multiple Inositol Polyphosphate Phosphatase as a contributor to aggregate soil phytase activity. HPLC also reveals the position of attack on phytate by different histidine phosphatases and affords opportunity for isolation of phytases for biotechnological use from other environments.

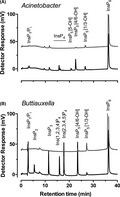

## Introduction

There are four forms of phytic acid (inositol hexakisphosphate, InsP_6_), which have been identified in nature, *myo*, *neo*‐, *scyllo*‐ and d‐*chiro*‐, that differ in their stereochemical conformation (Fig. S1) and association with metal ions as phytates in different soils (Turner *et al*., 2002). Among these, *myo‐*inositol hexakisphosphate (InsP_6_) garners the most attention from plant scientists. It is the principal storage form of phosphorus in plants, seeds and grains representing between 50% and 85% of the total phosphate in plants and forming as much as 1–5% of the dry weight in many seeds, grains and fruits (Raboy and Dickinson, 1993).

Monogastric animals such as swine and poultry are fed diets that are largely cereal‐ and/or grain‐based, but they lack sufficient levels of endogenous phytase, a mixed group of phosphatases that dephosphorylate phytate (Pandey *et al*., 2001). The undigested phytate and other ‘higher’ inositol phosphates are potent antinutrients by virtue of their ability to interfere with protein digestion and to chelate metal ions such as calcium, iron, magnesium, manganese and zinc, reducing their bioavailability. The first commercially produced phytase, *Natuphos*®, was released to the market in 1991 to improve the digestibility of grain phytate in the gastrointestinal tract of non‐ruminants (Lei and Porres, 2003). Since then, phytases have become a major sector of a global enzyme market of estimated value ca. $5 billion in 2015, with annual growth estimated at 6–8% from 2016 to 2020 (Guerrand, 2018).

Phytases are commonly separated into four categories, β‐propeller phytases (βPPhy), purple acid phytases (PAPhy), protein tyrosine phytases (PTPs) (cysteine phytases) and the histidine (acid) phosphatases (Mullaney and Ullah, 2007). The histidine (acid) phosphatases also comprise a subclass named multiple inositol‐polyphosphate phosphatases (MINPP) (Cho *et al*., 2006; Mehta *et al*., 2006; Haros *et al*., 2009; Tamayo‐Ramos et al., 2012; Stentz *et al*., 2014). The search for more effective enzymes – encompassing improved catalytic efficiency, protease stability , acid stability and thermo‐stability – and cost‐effective production has been extended to soil environments where *myo*‐, *neo*‐, *scyllo*‐ and d‐*chiro‐* forms of phytate represent substantial, albeit recalcitrant, ‘reserves’ of organic phosphate (Menezes‐Blackburn *et al*., 2018). The soil environment encompasses a diverse microflora, with estimates of 4000‐7000 different bacterial genomes per gram of soil (Ranjard *et al*., 2000). Consequently, soil has been a target for many phytase isolation efforts (Kumar *et al*., 2013; Puppala *et al*., 2019). Characterization of enzymes isolated from different environments has allowed comprehensive comparative analysis of stability and activity (Konietzny and Greiner, 2002; Mullaney and Ullah, 2003; Huang *et al*., 2006), aiding the development of thermo‐stable enzymes for industrial use (Lehmann *et al*., 2000; Wu *et al*., 2014). Nevertheless, several technical issues still frustrate efforts to identify and isolate phytase‐producing organisms, and assessment of their contribution to environmental turnover of organic phosphates, including phytate.

The small fraction of environmental organisms amenable to culture has the consequence that the biodiversity of phytase producers is grossly underestimated. Consequently, metagenomic and metaproteomic approaches have supplanted culture‐based approaches for study of the relationship of microbiological diversity and soil phosphorus (Neal *et al*., 2017; Yao *et al*., 2018; Chen *et al*., 2019). Alternatively, others have employed amplicon sequencing of functional phosphatases using *phoD* alkaline phosphatase‐specific primers (Ragot *et al*., 2015). When allied with heterologous expression, metagenomic methods have revealed novel catalytic diversity among phytate‐degraders extending classification beyond the four canonical classes (Castillo Villamizar *et al*., 2019a,b) as have more conventional functional genomic methods (Sarikhani *et al*., 2019).

Irrespective of the method of identification of candidate phytases, whether as commercial product leads or as contributors to environmental processes, both culture‐independent approaches and their culture‐dependent counterparts commonly rely on informative enzyme assays for characterization of the reactions catalysed. One issue with the assay most commonly used, phosphate detection with reagents such as molybdenum blue/malachite green, is the purity of the phytate substrate. Commercially available phytate is impure (Fig. S2A) and often contains substantial mole fractions of lower inositol phosphate and inorganic phosphate impurities (Nagul *et al*., 2015). Consequently, unless assays follow disappearance of phytate they risk measurement of pre‐existing inorganic phosphate or risk misidentification of enzymic activity towards ‘lower’ inositol phosphates. The literature offers historic precedent: isolates capable of degrading InsP_5_ but not InsP_6_ were identified in a seminal study of phytase isolation (Cosgrove *et al*., 1970).

The issue of substrate quality is relatively solvable; purification of InsP_6_ is well described (Cosgrove, 1980; Dorsch *et al*., 2003; Madsen *et al*., 2019), but rarely discussed in screening for phytases. ‘Phytase‐specific media’ (PSM) (Howson and Davis, 1983; Kerovuo *et al*., 1998) are used widely and rely on formation of clearing (of phytate precipitate) zones around bacterial colonies. The method suffers a high rate of false positives, arising from bacterial secretion of low molecular weight organic acids capable of solubilizing the phytate precipitates (Iyer *et al*., 2017). This itself highlights another issue with the approach – that it is not suitable for screening at low pH – a condition for which many commercial enzymes have been optimized. While solubilization may be overcome by a two‐step counterstaining test to re‐precipitate acid‐solubilized phytate (Bae *et al*., 1999), re‐precipitation does not indicate to what extent the available phytate has been degraded, since other ‘higher’ inositol phosphates can also be re‐precipitated. Autoclaving of the medium can also result in phytate degradation (see Fig. S2B,C) and resultant change to the pH of the media. Overall, clearing zones may not exclusively indicate enzymatic hydrolysis of phytate in the plate (Fredrikson *et al*., 2002), while pH limitations of the method will necessarily be selective of the organisms cultured. There is therefore opportunity for sensitive methodologies that allow characterization of the substrate and its utilization. Here, we adopt the PSM methodology and supplement it with high‐performance liquid chromatography (HPLC) to demonstrate a more accurate and quantitative method allowing screening and isolation of phytase‐producing organisms from environmental samples. We also show how different isolates produce different inositol phosphate profiles from phytate and extend the analysis to soil samples supplemented with phytate to follow the activity of aggregate cohorts of microbes. A schematic diagram of the range of analyses enabled is shown (Fig. S3).

## Results and discussion

### Acid extraction of phytate from PSM plates

**Fig. 1:**
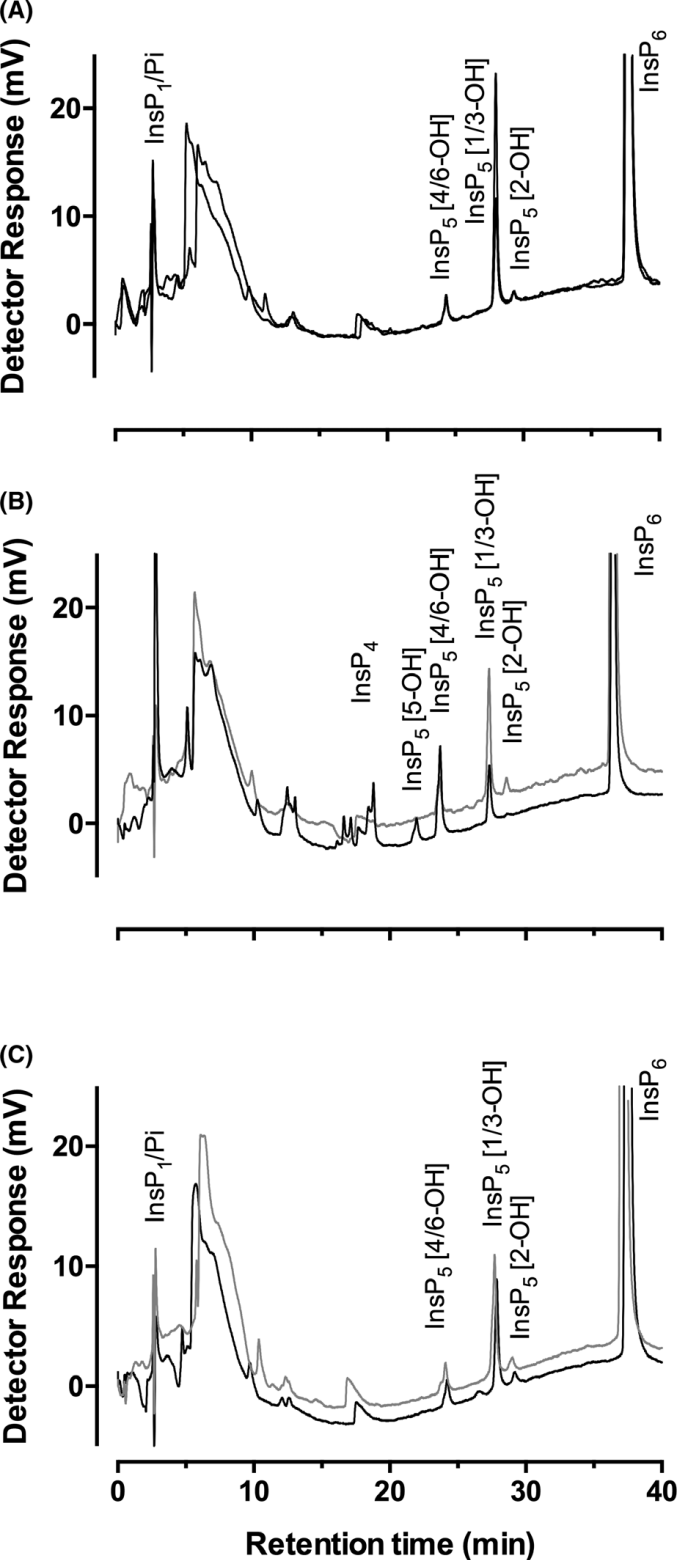
HPLC analysis of the inositol phosphate content of zones of agar of PSM‐grown bacteria. A. *Bacillus subtilis* ESKAPE strain; B. *Escherichia coli*‐pDES17‐*Btminpp* and C. *Pseudomonas putida* P450. A and C, non‐cleared agar, black lines; cleared agar, grey lines; B, non‐cleared agar, grey line. cleared agar, black line.

The PSM plate approach is one of the most commonly used methods for isolation of phytase‐positive organisms from soil, but it is not without the substantial drawbacks discussed above. Control strains of *Escherichia coli*–pDES17–*Btminpp* harbouring a plasmid‐borne MINPP from *Bacteroides thetaiotaomicron* (Stentz *et al*., 2014), *Bacillus subtilis* strain ESKAPE (predicted to contain βPPhy) and *Pseudomonas putida* J450 (predicted to contain βPPhy) were each streaked onto fresh PSM plates and allowed to grow over three days at 30°C. All isolates generated clearing zones around their biomass on these PSM plates. Cores of agar from ‘cleared’ and ‘non‐cleared, cloudy’ zones were extracted with HCl and the inositol phosphate profile thereof examined by HPLC (Fig. 1). While there can be slight differences in the efficiency of extraction between the cleared and cloudy zones, comparison of individual peaks within the respective profiles makes evident the different extents and pathways of phytate degradation by the strains.

All profiles from the ‘non‐cleared’ zones show the predominant peak of InsP_6_ with a retention time of *c*. 37 min and a smaller peak of InsP_5_ [1/3‐OH] contaminant with a retention time of *c*. 28 min, representing approximately 5% of total inositol phosphate in this ‘clean’ InsP_6_ substrate. Inorganic phosphate (Pi) elutes with the solvent front at *c*. 2.8 min. In respect of the ‘cleared’ zones, the *B. subtilis* strain ESKAPE (Fig. 1A) showed a small amount of InsP_6_ degradation with a small increase in InsP_5_ [1/3‐OH] and a concomitant increase in Pi. In this experiment, much of the InsP_6_ remained. The InsP_5_ [1/3‐OH] peak is the expected product of the known InsP_6_ D‐3‐phosphatase activity of the βPPhy (Kerovuo *et al*., 1998) originally characterized (Powar and Jagannathan, 1982). The *E. coli*‐pDES17‐*Btminpp* strain (Fig. 1B) showed considerably more activity, producing multiple peaks of InsP_5_, InsP_4_ and InsP_3_ intermediates, characteristic of MINPP enzyme (Haros *et al*., 2009; Tamayo‐Ramos et al., 2012; Stentz *et al*., 2014). There is also a larger Pi peak. Finally, the *P. putida* strain (Fig. 1C) showed little difference in the profile of ‘cleared’ *vs*. ‘non‐cleared’ agar despite the known InsP_6_ D‐3‐phosphatase activity of other *Pseudomonas* sp. (Cosgrove *et al*., 1970; Irving and Cosgrove, 1972).

Collectively, these comparisons demonstrate that zone clearing without careful normalization is a poor assay for phytate degradation even of well‐characterized organisms. It does illustrate however that HPLC can be combined with media‐based culture and extraction of agar for testing of phytate degradation to provide high sensitivity and diagnostic analysis of the likely enzyme activity, by the simple expedient of observation of the occurrence of InsP peaks not present in ‘non‐cleared’ regions of agar plates.

### Assay of phytate degradation by mixed population soil cultures

**Fig. 2:**
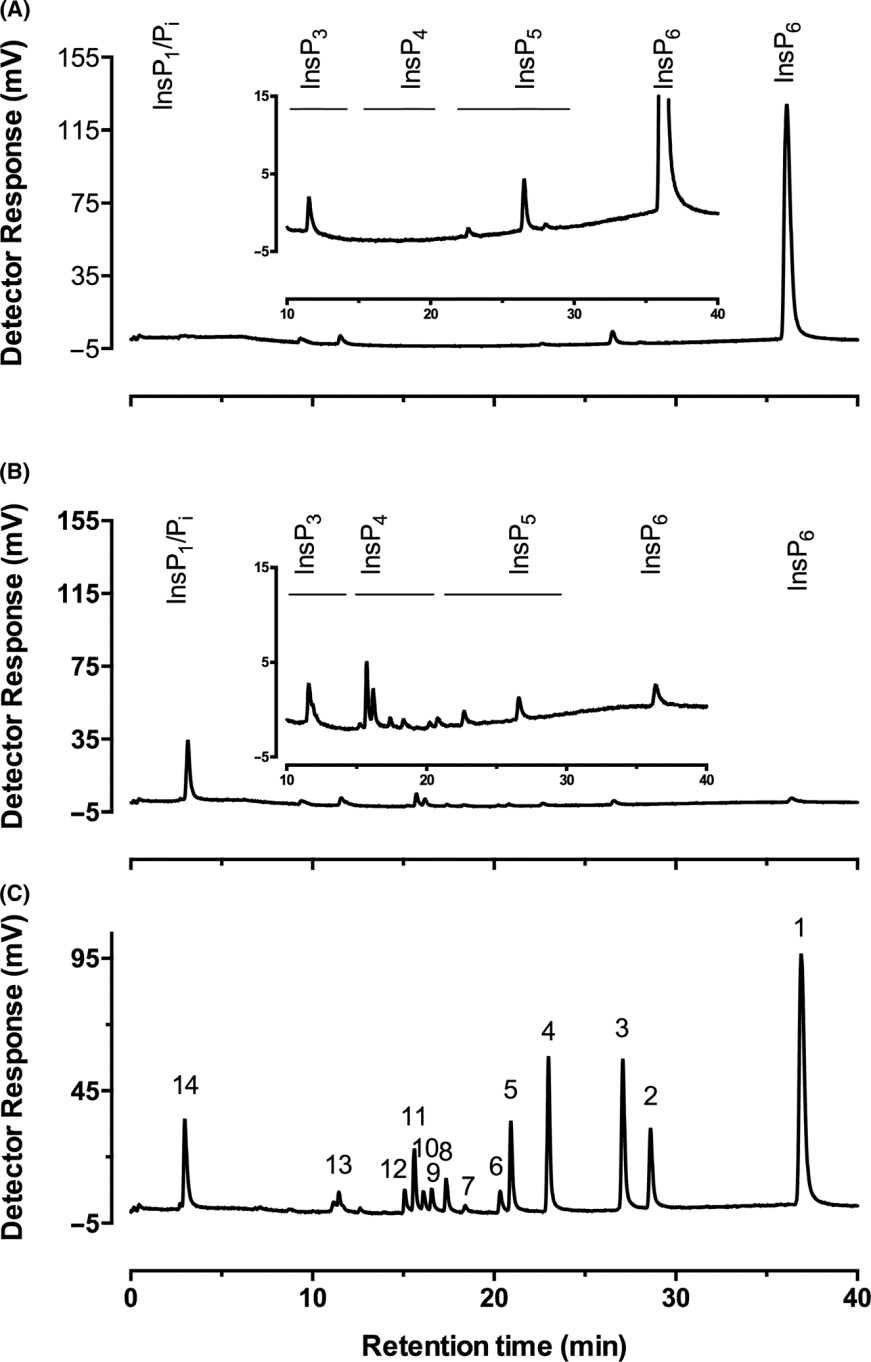
HPLC analysis of phytate degradation in liquid media of a soil culture. A. day 0, it shows minor impurities in the phytate substrate; B) by day 5, six InsP_4_ intermediates can be identified. Inset (A, B) shows traces expanded. C. A set of InsP standards prepared by acid reflux of phytate: the peaks identified are 1: InsP_6_, 2: InsP_5_ [2‐OH], 3: InsP_5_ [1/3‐OH], 4: InsP_5_ [4/6‐OH], 5: InsP_5_ [5‐OH], 6: InsP_4_ (1456/3456), 7: InsP_4_ (2456), 8: InsP_4_ (1256/2345), 9: InsP_4_ (1345/1356), 10: InsP_4_ (1245,2356), 11: InsP_4_ (1234/1236), 12: InsP_4_ (1246), 13: InsP_3_, and 14: InsP_1_/P_i_.

Phytate degradation may also be demonstrated with mixed cultures that might ordinarily be subjected to standard dilution and culture techniques for discrimination of individual isolates. In Fig. 2, we show the result of mixing soil with minimal medium containing InsP_6_ as the sole phosphate source. The soil was untilled (for the season) agricultural soil from Fakenham, Norfolk, UK, which we used to first test the technique. In this experiment, this agricultural soil was incubated with shaking at 30°C. Degradation of InsP_6_ was observed initially on day 3; by day 5 <5% of starting InsP_6_ remained, consistent with the accumulation of Pi, which co‐elutes with InsP_1_ on this column‐gradient method. The generation of multiple inositol phosphate peaks at all stages of dephosphorylation (InsP_5_, InsP_4_, InsP_3_ and InsP_2_) probably arises as a consequence of the action of several phytase enzymes, since the classification of phytases reflects predominant attack in discrete sequences and predominant accumulation of single InsP_5_ and InsP_4_ species.

**Fig. 3:**
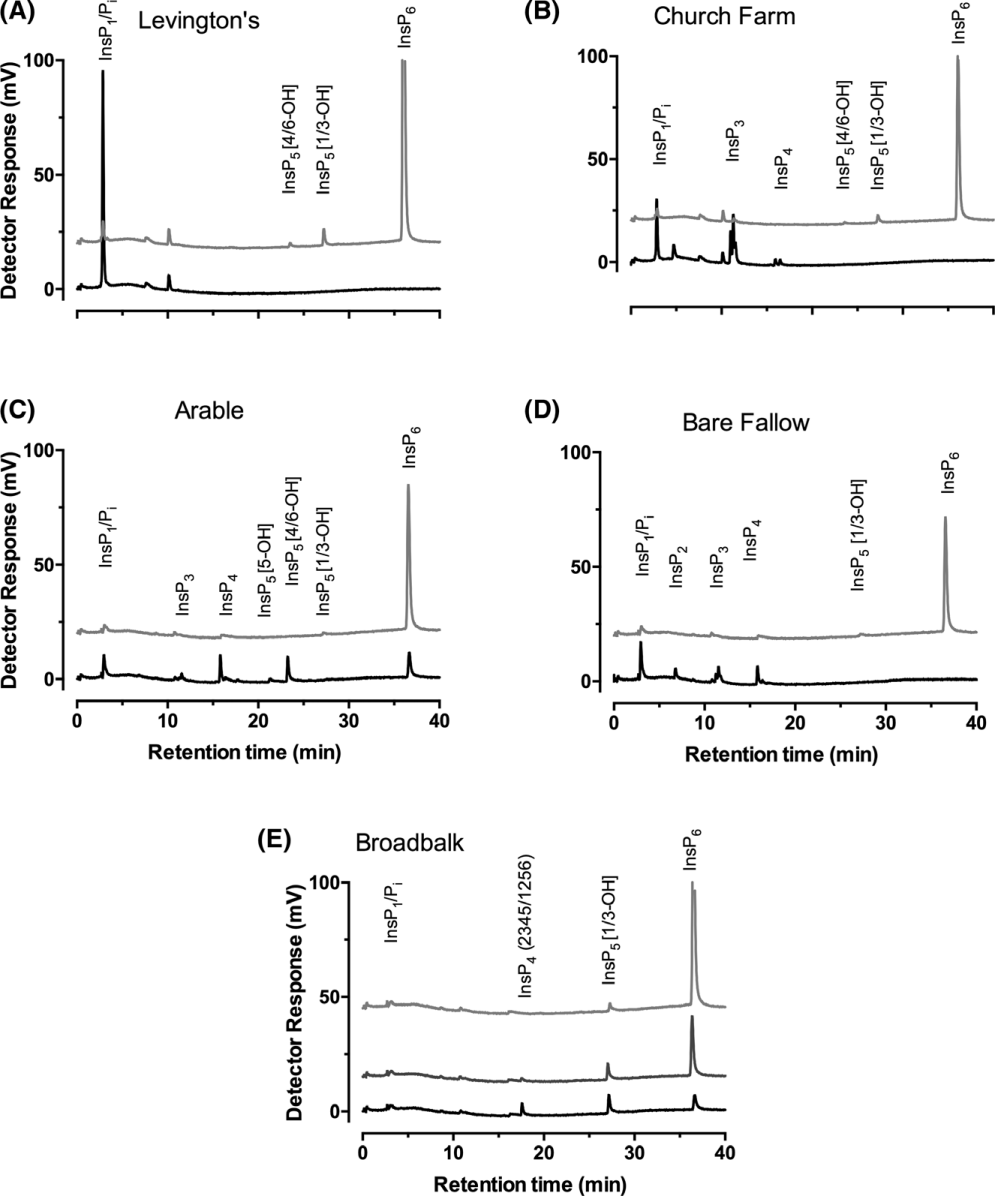
HPLC analysis of phytate degradation by five different soil matrices. A. Levington compost F2 and B. Church Farm were obtained in‐house, and C. Arable, D. Bare Fallow and E. Broadbalk were obtained from Rothamsted Research long‐term field experiments. For E, the soil suspension was supplemented with additional phytate. The traces are offset on the *Y*‐scale. Light grey lines, day 0 (A,B), day 1 (C,D) or day 3 (E). Black lines, day 2 (A–D), day 8 (E). Dark grey line, day 7 (E).

This experiment was repeated on five well‐characterized soil or plant growth matrices, all of which showed evidence of phytase activity. The first (Fig. 3A) is Levington compost F2, obtained from the John Innes Centre, Norwich, UK. The second (Fig. 3B) is soil sampled from Church Farm, the field study site of the John Innes Centre in Bawburgh, Norwich UK. The next three soils were sampled from two long‐term field experiments from Rothamsted Research, Harpenden, UK (Supporting information). The first sample (Fig. 3C) was obtained from continuous arable plots growing winter wheat (*Triticum aestivum* L.) of the Highfield Ley‐Arable Experiment. Also, from this site, soil was sampled from permanent bare fallow plots (Fig. 3E) that have been maintained crop‐ and weed‐free by regular tilling for over 50 years. The βPPhy genes in both these soils have been characterized by shotgun metagenomics (Neal *et al*., 2017). The gene sequences identified show homology to genes identified in *Bacillus*, *Paenibacillus*, *Alteromonas* and *Cyanothece* species. Soil was also collected from a plot of the Broadbalk Winter Wheat Experiment (Fig. 3D). Shotgun metagenome analysis of DNA extracted from this soil similarly showed the βPPhy gene sequences to be homologous to those in *Bacillus*, *Paenibacillus*, *Alteromonas* and *Cyanothece* (Neal and Glendining, 2019).

All the soil plant growth matrix types degraded phytate when added to liquid medium, generating distinct phytate degradation profiles and, but for one concomitant accumulation of inorganic phosphate (Fig. 3).

While degradation of phytate by some matrices, Levington’s compost (Fig. 3A), Church Farm (Fig. 3B) and Bare Fallow (Fig. 3D) proceeded to completion or close to it, indicated by predominant accumulations of Pi, other soils, which presumably had less abundant or active cohorts of microbes, yielded diagnostic InsP_5_ peaks in the timescale of the experiment. Of the Rothamsted soils, the Broadbalk soil removed phytate from liquid media such that neither phytate nor lower inositol phosphates were recovered, at day 0 (not shown). We attribute this to sorption of phytate to soil particles as this has up to 35% clay content. Nevertheless, by supplementing the soil/liquid mixture with 1 mM phytate we were subsequently able to show that the soil and associated microorganisms were capable of processing added phytate over 8 days (Fig. 3E). For this soil, Pi did not accumulate in the medium – suggesting that the microflora were efficiently scavenging the released phosphate.

### Classification of aggregate phytase activities of soil microbe populations

For phytases, the site of initial attack (Fig. S1) represents one ontology of enzyme classification. Enzyme Commission (EC) 3.1.3.26 – 4‐phytase – defines enzymes that remove phosphate from the 1D‐4 position of *myo*‐inositol hexakisphosphate (phytate) substrate (the original classification of this enzyme recognizes the detailed analysis of enantiomerism of phytate degradation products by cereal activities (Cosgrove, 1980 and Supplementary information), but the 3.1.3.26 signifier is commonly conflated with 1D‐6 phytase (1L‐4 phytase) activity. EC 3.1.3.8 – 3‐phytase – defines enzymes that remove phosphate from the 1D‐3 position. This distinction makes no consideration of structural fold or reaction mechanisms.

While bacterial and fungal [1d‐] 3‐phytases include histidine (acid) phosphatases with alpha‐beta and alpha domain structure, Rossmann fold and characteristic reaction mechanism involving an attacking histidine nucleophile (His) and proton donating acidic amino acid (Oh *et al*., 2004; Mullaney and Ullah, 2007), some [1d‐] 3‐phytases possess different structural folds. The βPPhy, exemplified by the enzyme from *B. amyloliquefaciens*, is a calcium‐dependent metalloprotein with catalytic and structurally important bound Ca^2+^ ions (Shin *et al*., 2001). Equally, histidine phosphatases include enzymes (phytases) that attack the 1D‐6 position, exemplified by AppA from *E. coli* (Greiner *et al*., 1993), *Buttiauxella* sp. (Cervin et al., 2008), *Citrobacter* sp. (Kim *et al*., 2003; Pontoppidan *et al*., 2012) and *Hafnia alvei* (Ariza *et al*., 2013). These are classified as EC 3.1.3.2 acid phosphatases. A comprehensive review of histidine phosphatases (Rigden, 2008) places fungal phytases and bacterial acid phosphatases in a branch of a superfamily of functionally diverse histidine phosphatases, which include the enzymes phosphoglycerate mutase and fructose‐2,6‐bisphosphatase.

Because simple HPLC resolves the two *meso*‐compounds, InsP_5_ [2‐OH] and InsP_5_ [5‐OH] from the enantiomeric pairs InsP_5_ [1/3‐OH] and InsP_5_ [4/6‐OH] (Fig. 2C), it is easy to distinguish between 4‐phytases (EC 3.1.3.26) and 3‐phytases (EC 3.1.3.8) or acid phosphatases (EC 3.1.3.2). For example, comparison of Highfield arable soil (Fig. 3C) – which yielded predominantly InsP_5_ [4/6‐OH] – with soil from a plot from Broadbalk (Fig. 3E) – which yielded InsP_5_ [1/3‐OH] – indicates that the dominant contributors to phytate degradation in our assays are different enzymes. They probably represent 6‐dephosphorylating histidine (acid) phosphatase (phytase) of EC 3.1.3.2 acid phosphatase (Highfield) and 3‐dephosphorylating βPPhy classes (Broadbalk) (Neal and Glendining, 2019). The absence of InsP5 [5‐OH], diagnostic for EC 3.1.3.72 – 5‐phytase – and exemplified by lily pollen alkaline phosphatase (Barrientos *et al*., 1994), a eukaryotic MINPP (Mehta *et al*., 2006), *Bifidobacterium pseudocatenulatum* MINPP (Haros et al., 2009) and *Bacteroides thetaiotaomicron* MINPP (Stentz *et al*., 2014), precludes dominant contribution from these classes of enzyme. Of course, while generation of a peak of, e.g., InsP_5_ [4/6‐OH] could arise from attack at the [1d‐] 6‐position by an *E. coli*‐like histidine (acid) phosphatase or from attack at the [1d‐] 4‐position by an enzyme with similar activity to the cereal phytase, the inclusion of cycloheximide in our media prevents eukaryotic growth. The situation is further compounded by the first report of a bacterial PAPhy with predominant InsP_5_ [4/6‐OH] product, harboured by a soil earthworm cast microbe with similarity to *Sphingobium yanoikuyae* (Nasrabadi *et al*., 2018).

### Identification of Acinetobacter and *Buttiauxella* sp. in soil samples

In an extension to the above analyses, we amplified and sequenced 16S rRNA genes from isolates of agricultural and Church Farm soils giving HPLC profiles of Figs 2 and 3B. These isolates designated AC1‐2 and CH‐10‐6‐4 (both from 10^6^ dilutions of these soil samples) are both *Gammaproteobacteria*. The amplified 16S rRNA gene of strain AC1‐2 (MT450216) was identical to that of *Acinetobacter* sp. strain YAZ49, *Acinetobacter calcoaceticus* strain EB11, *Acinetobacter calcoaceticus* strain P19 and that recovered from whole‐genome sequencing (JABFFO000000000) of the parent isolate AC1‐2. The 16S rRNA gene of strain CH‐10‐6‐4 was identical to that in *Buttiauxella agrestis* strain EB112, *Buttiauxella* sp. CL_136_AN_40 and *Buttiauxella* sp. SA_136_AN_45 (MT450213‐MT450215). To investigate the distribution of different phytases in these two genera, BLAST searches were conducted using ratified examples of each of five phytase families, restricting searches only to *Acinetobacter* and *Buttiauxella* species (Table 1). For PAPhy, we followed Nasrabadi *et al*. (2018) using *Lupinus luteus* (AJ505579) as query to blast *Sphingobium* spp. genomes, returning hits with percentage identity 23‐26% with E value of 5^‐12^ to 9^‐10^ . Subsequently, the full gene from *Sphingobium yanoikuyae* (CP060122) was used as query of *Acinetobacter* and *Buttiauxella* spp. These searches indicated a divergent distribution of phytase families between the two organisms. *Acinetobacter* species were predominantly associated with MINPP and βPPhy, while histidine (acid) phosphatase was the only phytase family associated with *Buttiauxella* species.

**Table 1:** Frequency of canonical phytase classes between referenced genomes (Protein Blast of Non‐Redundant Protein Sequences at NCBI) of *Acinetobacter* and *Buttiauxella* spp.

	*Acinetobacter* spp.	*Buttiauxella* spp.
MINPP	445	0
HAP	9	24
βPPhy	80	0
PTP	0	0
PAPhy	1	0

The reference sequences used were as follows: multiple inositol‐polyphosphate phosphatase (MINPP), *Bacteroides thetaiotaomicron* (WP_009040027); histidine (acid) phosphatase (HAP), *Citrobacter amalonaticus* (DQ975370.1; β‐propeller phytase (βPPhy), *Bacillus amyloliquefaciens* (WP_013352583); protein tyrosine phosphatase (PTP), *Selenomonas lacticifex* (ABC69367) and Purple Acid Phytase (PAPhy), *Sphingobium yanoikuyae* (CP060122).

To interrogate further the phylogenetic separation of histidine (acid) phosphatase between *Acinetobacter* spp. and *Buttiauxella* spp., revealed in Table 1, a diverse selection of accessions (reference genomes) of each sp. was searched by tblastn in NCBI with the different phytase reference sequences of Table 1 as query. The results are shown in Table S1, in which crosses indicate the presence of the different phytase proteins in selected genome‐sequenced *Acinetobacter* and *Buttiauxella* strains yielding *E* value < 0.00005. Only a single histidine (acid) phosphatase was present in the *Buttiauxella* genomes analysed. These were either AppA phytases or bifunctional glucose‐1‐phosphatase/inositol phosphatases. The phytase complements of *Acinetobacter* genomes were more varied, revealing the presence of all different classes of phytase with the exception of protein tyrosine phosphatase. Additionally, while predominantly only containing a single phytase, there were some cases of *Acinetobacter* sp. containing two different classes of phytase: either MINPP and βPPhy, or histidine (acid) phosphatase and βPPhy.

### Phytate degradation profiles of isolated *Acinetobacter* and *Buttiauxella* strains reveal distinct histidine phosphatase activities

**Fig. 4:**
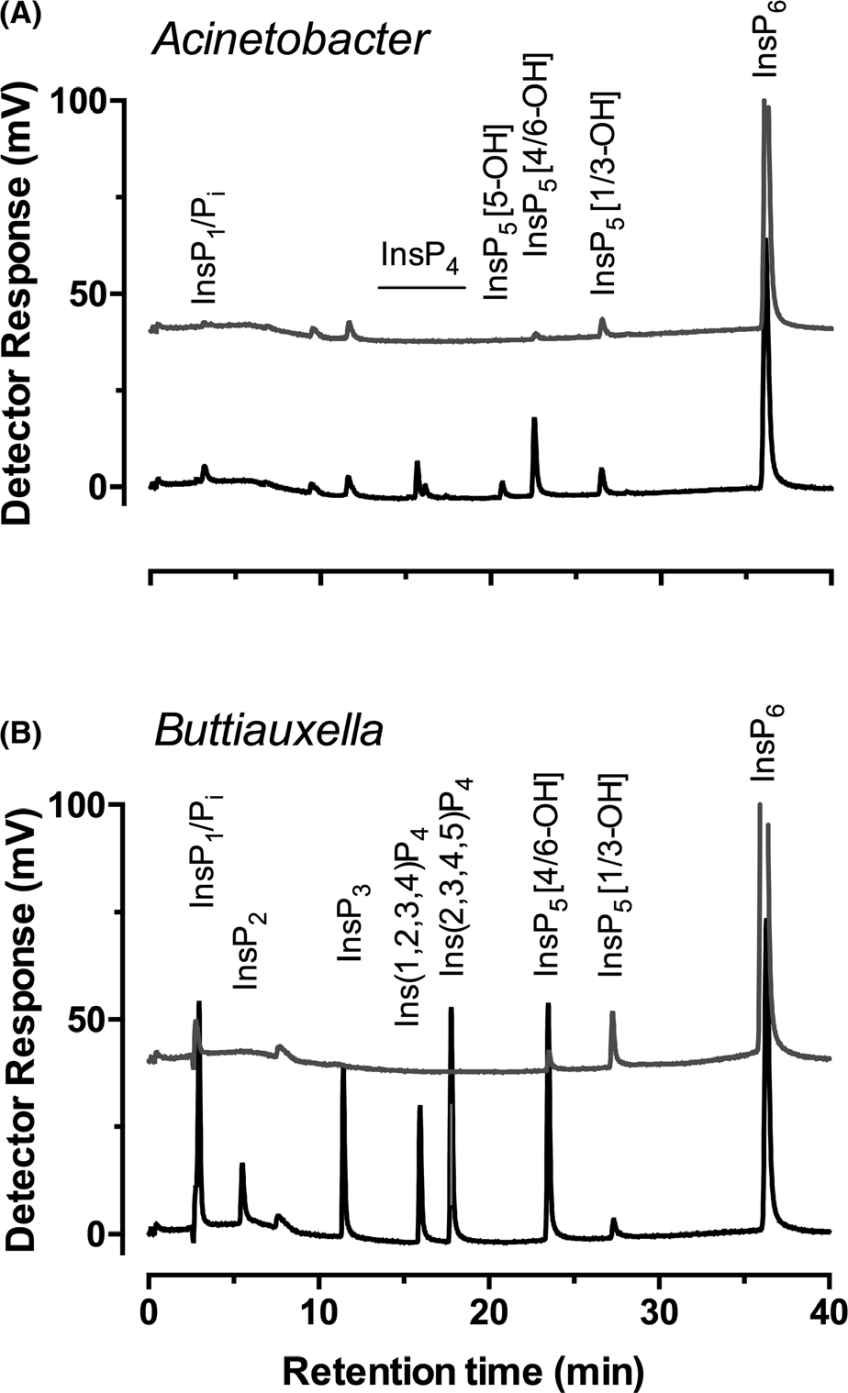
HPLC analysis of example phytate‐degrading isolates. A. isolate AC1‐2 (*Acinetobacter*) from agricultural soil and B. isolate CH‐10‐6‐4 (*Buttiauxella*) from Church Farm. Grey lines, day 0; black lines, day 2.

To confirm the ability of identified isolates bearing defined cohort(s) of phytase(s) to degrade phytate and to characterize those enzyme activities, the isolates *Acinetobacter* sp. AC1‐2 (AC1‐2) and *Buttiauxella* sp. isolate CH‐10‐6‐4 were incubated with phytate and subjected to HPLC analysis (Fig. 4A,B). This demonstrated that enzymes associated with AC1‐2 are promiscuous in their site of initial attack on phytate substrate, yielding among InsP_5_ isomers a dominant 4/6‐OH peak, a smaller 5‐OH peak and little to no detectable degradation at the 1/3‐position (Fig. 4A). Interestingly, strain CH‐10‐6‐4 did not show any phytase activity in minimal medium, but it did however degrade 1 mM phytate when incubated in a 20 mM Tris–HCl and 0.1% NaCl solution (Fig. 4B).

The *Buttiauxella* strain CH‐10‐6‐4 (Fig. 4B) showed a high specificity towards the initial position of attack on phytate, generating InsP_5_ [4/6‐OH] predominantly among InsP_5_ products, consistent with the published properties of *Buttiauxella* phytase (Cervin et al., 2008) and its industrial use (Ushasree *et al*., 2017; Herrmann *et al*., 2019).

While both the *Acinetobacter* and *Buttiauxella* strains showed preferential 1D‐4/6 selectivity of attack on phytate, they differ in terms of the resulting InsP_4_ intermediates: the *Acinetobacter* strain produced four InsP_4_ intermediates, while the *Buttiauxella* strain produced two, a predominant peak with the chromatographic properties of d/l‐Ins(2,3,4,5)P_4_ and a minor peak with that of d/l‐Ins(1,2,3,4)P_4_. Again, HPLC can be shown to distinguish between classes of phytase without assistance of 16S rRNA gene. The phytate degradation profile of the *Buttiauxella* isolate is characteristic of 1D‐6‐directed histidine (acid) phosphatase, that of the *Acinetobacter* strain was indicative of the MINPP subclass of the histidine (acid) phosphatases (Tamayo‐Ramos et al., 2012; Stentz *et al*., 2014). Congruent with these predictions, strain CH‐10‐6‐4 was shown by PCR to contain a histidine (acid) phosphatase, 100% identical at the amino acid level to that in *Buttiauxella ferragutiae*. Furthermore, the genome sequence of AC1‐2 was shown to encode a MINPP 98.28% identical at amino acid level to that in *Acinetobacter calcoaceticus*.

**Fig. 5:**
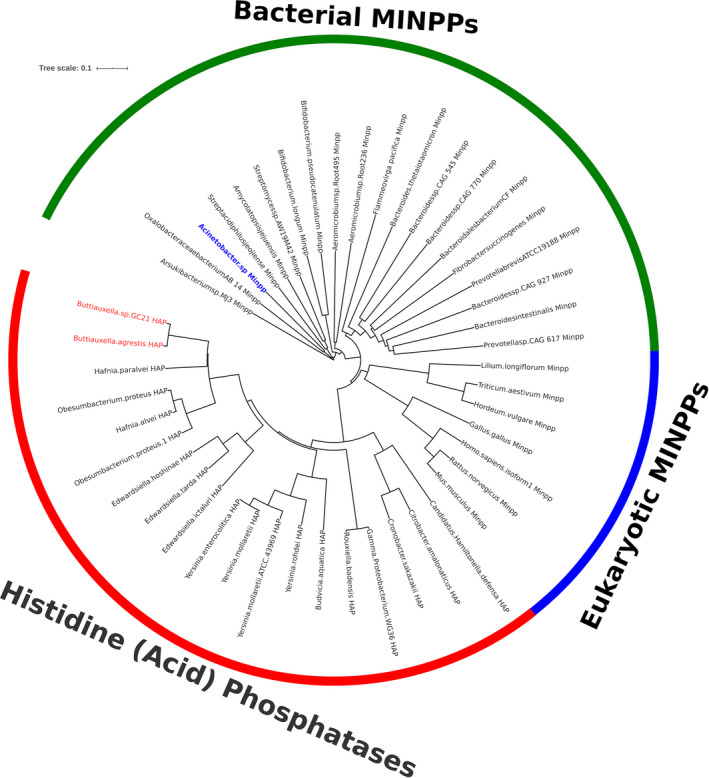
Phylogram of thirty‐one histidine acid phosphatases and twenty‐seven multiple inositol‐polyphosphate phosphatases (MINPP) showing the evolutionary differences between the two sets of genes. The *Acinetobacter* sp. gene sequenced (JABFFO000000000) is highlighted in blue. The *Buttiauxella* strain highlighted in red is a species similar to that identified by 16S RNA sequencing of CH‐ CH‐10‐6‐4 (accession MT680195).

With this additional information, we undertook an alignment of phytase protein sequences for thirty‐one histidine (acid) phosphatases and twenty‐seven MINPPs using the online multisequence alignment tool MAFFT (Katoh *et al*., 2019), reporting the output as an Interactive Tree of Life, iTOL (Letunic and Bork, 2019) (Fig. 5). The results of this analysis split MINPP sequences into two clades, those whose origins are from animals and plants (Cho *et al*., 2006; Dionisio *et al*., 2007), and those from bacteria (Haros *et al*., 2009; Tamayo‐Ramos et al., 2012; Stentz *et al*., 2014). Both are distinct from bacterial histidine (acid) phosphatases, with bacterial MINPPs more closely related to eukaryotic MINPPs than bacterial histidine (acid) phosphatases. Of the bacterial MINPPs, the *Acinetobacter* enzyme was more deeply rooted than the MINPPs of previously characterized gut commensals *Bifidobacter* and *Bacteroides* spp.

### Improved, predictive HPLC‐based screening for phytases

The foregoing analyses highlight considerations that apply to culture‐dependent isolation of phytases, here from environmental samples. The methods described overcome problems associated with the purity of phytate substrate (Madsen *et al*., 2019) and ‘zone‐clearing’ assays (Fredrikson *et al*., 2002). Nevertheless, PSM can be a useful media for obtaining a diverse set of bacteria (Greiner *et al*., 1997; Richardson & Hadobas, 1997; Kerovuo *et al*., 1998) or for the screening of engineered bacteria and plants (Shulse *et al*., 2019).

Here, the opportunity to characterize enzyme activity of isolates before functional cloning, expression, purification, subsequent verification of catalytic activity is a considerable shortcut that focuses attention among isolates on those with *bona fide* phytase activity. Moreover, sequencing of the *Acinetobacter* and *Buttiauxella* strains revealed the power of this HPLC‐based screening strategy to illuminate phytase diversity. The two different histidine phosphatases, MINPP and histidine (acid) phosphatase, are typical of the families of enzymes identified in sequenced genera. The assembled sequenced genome (JABFFO000000000) of the *Acinetobacter* strain AC1‐2 harbours a single histidine (acid) phosphatase of the MINPP class, rather than a canonical histidine (acid) phosphatase.

The enzyme bears a hepta‐peptide catalytic site sequence motif of RHGSRGL: RHG is characteristic of the histidine phosphatase superfamily (Rigden, 2008), and the proton donor motif is HAE, with glutamate replacing aspartate of the HD motif of histidine (acid) phosphatases. AC1‐2 MINPP is more closely related to eukaryotic, plant and animal MINPP than it is to bacterial histidine (acid) phosphatases. Significantly, the only prior functional identification of a bacterial MINPP is that of the human gut commensals *Bifidobacterium pseudocatenulatum* and *longum* subsp. *infantis* (Haros *et al*., 2009; Tamayo‐Ramos et al., 2012) and *Bacteroides thetaiotaomicron* (Stentz *et al*., 2014) that share the HAE motif. Other homologues can be found among the *Actinobacteria*, *Betaproteobacteria* and *Gammaproteobacteria* (Tamayo‐Ramos et al., 2012; Stentz *et al*., 2014). Our identification of significant contribution of MINPP to aggregate environmental phytase activity and to *Acinetobacter*, particularly, serves to highlight novel biotechnological opportunity of exploitation of environmental samples. *Acinetobacter* spp. are commonly cited in context, but in no means as the principal agent, of enhanced biological phosphorus removal (Seviour *et al*., 2003). They harbour a polyphosphate kinase *ppk* that is induced by Pi starvation (Trelstad *et al*, 1999). It seems likely therefore that the function of MINPP may be related to Poly P accumulation in soil *Acinetobacter*.

The second isolate was identified as a *Buttiauxella* strain, and comparison with published genomes of similar strains revealed, in contrast, a single canonical histidine (acid) phosphatase. BLAST searches of *Buttiauxella* accessions for all phytase classes yielded only histidine (acid) phosphatase with E values less than 10^‐68^. These were of the *E. coli* AppA family histidine acid phosphatase (Lim *et al*., 2000) with RHGVRAP and HDTN motifs, or bifunctional glucose 1‐phosphatase/phytase (Golovan *et al*., 2000; Lee *et al*., 2003) class with RHNLRAP (similar to RANLRAP (Lee *et al*., 2003)) and HDSN (similar to HDQN (Lee *et al*., 2003)) motifs. The *Buttiauxella* sp. AppA and its engineered variants (Cervin et al., 2008) are already a commercial product used widely to improve pig and poultry performance (e.g. Adedokun *et al*., 2015). Other bacterial AppA enzymes, e.g., from *E. coli* and *Citrobacter* spp., are used similarly (Sommerfeld *et al*., 2018; da Silva *et al*., 2019). Our unbiased, for phytase class, screening approach is clearly capable of identifying candidate phytases with potential as commercial leads.

## Experimental procedures

### Media

Agar was obtained from Sigma (Merck Life Science UK Limited, Dorset, UK). Tryptone and yeast extract for preparation of Lysogeny broth were obtained from Formedium (UK).

### Preparations of soil cultures

Soil (0.5 g) was added to 10 ml of minimal media, pH 7, in a 30 ml universal. The base media, modified from Neal *et al*.(2017), comprised: 18.7 mM NH_4_Cl, 8.6 mM NaCl, 1 mM MgSO_4_, 0.1 mM CaCl_2_, 1 mM succinate, 1mM glucose, 1mM sucrose, 1mM pyruvate, pH 7 and 1 mM InsP_6_. The media were supplemented with vitamins: 10 µl of vitamin solution (containing 10 mg pyridoxine.HCl, 5 mg thiamine.HCl, 5 mg riboflavin, 5 mg para‐amino benzoic acid, 5 mg nicotinic acid, 2 mg vitamin B12, 2 mg folic acid, l^‐1^) and with micronutrients: 10 µL (2 g nitriloacetic acid, 1 g MnSO_4_.6H_2_O, 0.8 g Fe(NH_4_)_2_(SO_4_)_2_, 0.2 g CoCl_2_.6H_2_O, 0.2 g ZnSO_4_.7H_2_O, 20 mg CuCl_2_.2H_2_O, 20 mg NiCl_2_.6H_2_O, Na_2_MoO_4_.2H_2_O l^‐1^). The medium included 0.1‐0.2 mg ml^‐1^ cycloheximide to inhibit fungal growth. Soil suspensions were incubated under shaking at 180 RPM and 30 °C for six days, taking samples each day. Samples were diluted and plated onto LB media and incubated for 2 days at 30°C.

### Acid extraction of phytate from phytase‐specific media plates

Bacterial cells were washed off the plate using dH_2_O, and 100 mg samples of agar were extracted with 400 µl 0.8 M HCl with vortexing after disruption of the agar with a plastic stirrer. Samples were extracted for 15 min at room temperature and centrifuged at 13 000 *g* for one minute. The supernatant was removed with a HPLC needle and syringe and filtered through a 13 mm diameter 0.45 µm pore PTFE syringe filter (Kinesis, UK) into a borosilicate glass HPLC vial (Chromacol 03‐FISV(A)).

### Preparation of Soil Cultures for HPLC Analysis

Five hundred µl of a well‐mixed soil culture in media was centrifuged at 13 000 *g* for 5 min. The supernatant was filtered through a 13 mm diameter 0.45 µm pore PTFE syringe filter (Kinesis) and centrifuged again, and an aliquot (200 µl) was dispensed into an HPLC vial.

### HPLC Analysis of Inositol Phosphates

Inositol phosphates were analysed according to Whitfield *et al*. (2018).

Chromatography data were exported as *x*,*y* data and redrawn in GraphPad Prism v.6.0 (GraphPad Software, San Diego, CA, USA).

### 16S amplification

Single bacterial colonies were purified, and their 16S rRNA gene was amplified using the primers 28F (5′‐GAGTTTGATCNTGGCTCAG‐3′) and 519R (5′‐GWNTTACNGCGGCKGCTG‐3′) from genomic DNA using colony PCR. The PCR generated a single band resolved on a 1 % agarose gel, and this was purified using a QIAquick Gel Extraction Kit (QIAGEN). Sequencing of these PCR products at Eurofins (MWG, Ebersberg, Germany) identified the two isolates further examined in this study as strains of *Acinetobacter* sp. and *Buttiauxella* sp. To confirm that the isolated *Buttiauxella* sp CH‐10‐6‐4 contained a histidine (acid) phosphatase, primers were designed to the *appA* gene using sequenced *Buttiauxella* spp. genomes (*Buttiauxella* sp. JUb87, *Buttiauxella* sp. A111, *Buttiauxella agrestis*, *Buttiauxella ferragutiae*, *Buttiauxella brennerae*, *Buttiauxella gaviniae*, *Buttiauxella noackiae*, *Buttiauxella* sp. BIGb0552, *Buttiauxella* sp. 3AFRM03). These primers (Forward 5′‐GCG AGA ART TTC AAC ARC AGG −3′, Reverse 5′‐GTG YCC GGC AAK AAA CAG G‐3′) were used to amplify a 725‐bp product from the *Buttiauxella* sp. isolate. These PCR products were sequenced by Eurofins, and their identity to ratified *Buttiauxella* spp. *appA* genes was established by BLAST analysis. The sequence was deposited in GenBank under the accession MT680195.

### Sequencing of strains

The *Acinetobacter* sp. strain AC1‐2 genome was sequenced by MicrobesNG (University of Birmingham, UK) using Illumina technology. This Whole Genome Shotgun Project has been deposited at DDBJ/ENA/GenBank under the accession JABFFO000000000. The version described in this paper is version JABFFO010000000. Genomic completeness was analysed using BUSCO v3 (Simao *et al*., 2015), an open‐source software that provides quantitative measures for genomic completeness based on evolutionarily informed expectations of gene content from near‐universal single‐copy orthologs selected. The *Acinetobacter* sp. strain AC1‐2 completeness was measured at 98 and 98.9% from both BUSCO’s bacterial and *Gammaproteobacteria* databases, respectively.

## Conflict of interest

None declared.

## Author contributions

GDR performed experiments, curated data and provided an original draft. JDT supervised experiments and edited the manuscript. ALN supervised experiments, curated data and wrote the manuscript. CAB secured funding, supervised experiments and wrote the manuscript.

## Supporting information


**Fig. S1**. Naturally occurring forms of inositol hexakisphosphate (phytate).
**Fig. S2**. Purity of commercial phytate.
**Fig. S3**. Schematic of workflow.
**Table S1.** Canonical phytase complements of referenced *Acinetobacter* and *Buttiauxella* genomes summarized in Table 1.
