# Supplementary material for: Improved sensitivity, accuracy and prediction provided by a high‐performance liquid chromatography screen for the isolation of phytase‐harbouring organisms from environmental samples
Source: Microb Biotechnol. 2020 Dec 21;14(4):1409–21. doi: 10.1111/1751-7915.13733 (PMC8313252; doi:10.1111/1751-7915.13733)
Supplement: Supplementary file 1 — Fig. S1. Naturally occurring forms of inositol hexakisphosphate (phytate). Fig. S2. Purity of commercial phytate. Fig. S3. Schematic of workflow. Table S1. Canonical phytase complements of referenced Acinetobacter and Buttiauxella genomes summarized in Table 1. [file MBT2-14-1409-s001.docx]

Improved sensitivity, accuracy and prediction provided by a High-Performance Liquid Chromatography screen for the isolation of phytase-harbouring organisms from environmental samples.

Running title: MINPP activity in soil-dwelling *Acinetobacter*

Gregory Rix^1^, Jonathan D. Todd^1^, Andrew L. Neal^2^ and Charles A. Brearley^1^

^1^ School of Biological Sciences, University of East Anglia, Norwich Research Park, Norfolk NR4 7TJ; ^2^ Department of Sustainable Agriculture Science, Rothamsted Research, North Wyke, Devon EX20 2SB.

Supporting Information:

Nomenclature

Classification of phytases: seminal literature to Enzyme Commission classifications

Characteristics of soils studied

Figure S1: Naturally occurring forms of inositol hexakisphosphate (phytate)

Figure S2: Purity of commercial phytate

Figure S3: Schematic of workflow

Table S1: Canonical phytase complements of referenced *Acinetobacter* and *Buttiauxella* genomes summarized in Table 1.

Nomenclature

The term ‘Ins’ with prefix 1D-*chiro*-, *myo*-, *neo*- or *scyllo*- is used as an abbreviation of the described inositol phosphate. Hence, *myo*-inositol 1,2,3,4,5,6-hexakisphosphate is abbreviated *myo*-Ins(1,2,3,4,5,6)P_6_, or, since this molecule is unique, *myo*-InsP_6_. However, because *myo*-InsP_6_ is the only stereoisomer of inositol hexakisphosphate whose breakdown products are readily available to researchers and is the only stereoisomer used in this study, we use the term InsP_n_ to signify a *myo*-inositol phosphate with n phosphates. According to the relaxation of rules for numbering of carbons in *myo*-inositol (IUB, 1989) we use the ‘1D-‘ numbering convention, with or without the ‘1D-‘ prefix, but for InsP_5_s, e.g., Ins(2,3,4,5,6)P_5_, we also use the shorthand form InsP_5_ [1-OH]. Where chromatography does not allow resolution of enantiomers e.g., Ins(2,3,4,5,6)P_5_ and Ins(1,2,4,5,6)P_5_, we use the term InsP_5_ [1/3-OH]. For co-eluting enantiomers of InsP_4_ e.g., [1D-]Ins(1,4,5,6)P_4_ and [1D-]Ins(3,4,5,6)P_4_, we also use the term InsP_4_ (1456/3456) in figure legends. We note that the numbering of phosphate substituents (of the carbon atoms to which they are attached) is not the same for all stereoisomers of inositol. The reader is referred to (Thomas et al., 2016) for a comprehensive review of inositol and inositol phosphate nomenclature and terminology.

Thomas, M.P., Mills, S.J., Potter, B.V.L. 2016. The "other" inositols and their phosphates: synthesis, biology, and medicine (with recent advances in *myo*-inositol chemistry). *Angew Chem Int Ed Engl* **55**: 1614-1650.

Enzyme Commission Classification of Phytases

Cosgrove, D.J. 1980. Inositol Phosphates: Their Chemistry, Biochemistry and Physiology. Elsevier, Amsterdam.

Irving, G.C., Cosgrove, D.J. 1972. Inositol phosphate phosphatases of microbiological origin: the inositol pentaphosphate products of *Aspergillus ficuum* phytases. *J Bacteriol* **112**(1): 434-8.

Johnson, L.F., Tate., M.E. 1969. The structure of *myo*-inositol pentaphosphates. *Ann NY Acad Sci* **165**: 526-532.

Lim, P.E., Tate, M.E. 1973. The phytases. II. Properties of phytase fractions F1 and F2 from wheat bran and the *myo*-inositol phosphates produced by fraction F2. *Biochim Biophys Acta* **302**(2): 316-28.

McDonald, A.G., Tipton, K.F. 2014. Fifty-five years of enzyme classification: advances and difficulties. *FEBS J* **281**(2): 583-92.

Tomlinson, R.V., Ballou, C.E. 1962. *Myo*-inositol polyphosphate intermediates in the dephosphorylation of phytic acid by phytase. *Biochemistry* **1**: 166-71.

Webb, E.C. 1992. *Enzyme nomenclature 1992: recommendations of the Nomenclature Committee of the International Union of Biochemistry and Molecular Biology on the nomenclature and classification of enzymes.* Academic Press.

Soil parameters

Levington Compost F2: pH 5.3 – 5.7 and N: P: K, 150:200:200 mg L^-1^ (Creissen et al., 2016).

Church Farm, the field study site of the John Innes Centre in Bawburgh, Norwich UK: pH of 7.5 and N: P: K: Mg, 149:171:184:60 mg Kg^-1^ (Turner et al., 2013).

Rothamsted Research , Harpenden, UK.

Soil was sampled from the Continuous Arable plots growing winter wheat (*Triticum aestivum* L.) of the Highfield Ley-Arable experiment (Gregory et al., 2009) (Figure 3C). The soil receives ammonium nitrate fertilisation to provide approximately 220 kg-N ha^−1^ annum^−1^, and additional 250 kg-K ha^−1^ and 65 kg-P ha^−1^ every three years: it has been cultivated for over 60 years. Also, from this site, soil was sampled from permanent Bare Fallow plots (Figure 3E) that have been maintained crop‐ and weed‐free by regular tilling for over 50 years. Soil was also collected from a plot of the Broadbalk Winter Wheat Experiment, which began in 1844 (Goulding et al., 2000) (Figure 3D). The samples were collected from a plot that has received inorganic fertilization of 192 kg-N ha^-1^, (96 kg-N ha^-1^ 1906–2000), 90 kg-K ha^-1^ and 12 kg-Mg ha^-1^ annum^-1^ since 1906, but has never received phosphorus fertilization. Measurement of Olsen-P in this soil only began in 1966, but has remained consistently below the estimated starting Olsen-P of 10 mg-P kg^−1^ (Neal & Glendining, 2019).

Creissen, H.E., Jorgensen, T.H., Brown, J.K.M. 2016. Impact of disease on diversity and productivity of plant populations. *Functional Ecology* **30**: 649-657.

Goulding, K.W.T., Poulton, P.R., Webster, C.P., Howe, M.T. 2000. Nitrate leaching from the Broadbalk Wheat Experiment, Rothamsted, UK, as influenced by fertilizer and manure inputs and the weather. *Soil Use and Management* **16**: 244-250.

Gregory, A.S., Watts, C.W., Griffiths, B.S., Hallett, P.D., Kuan, H.L., Whitmore, A.P. 2009. The effect of long-term soil management on the physical and biological resilience of a range of arable and grassland soils in England. *Geoderma* **153**: 172-185.

Neal, A.L., Glendining, M.J. 2019. Calcium exerts a strong influence upon phosphohydrolase gene abundance and phylogenetic diversity in soil. *Soil Biology and Biochemistry* **139**: 10.1016/j.soilbio.2019.107613

Turner, T.R., Ramakrishnan, K., Walshaw, J., Heavens, D., Alston, M., Swarbreck, D., Osbourn, A., Grant, A., Poole, P.S. 2013. Comparative metatranscriptomics reveals kingdom level changes in the rhizosphere microbiome of plants. *ISME J* **7**: 2248-2258.

**
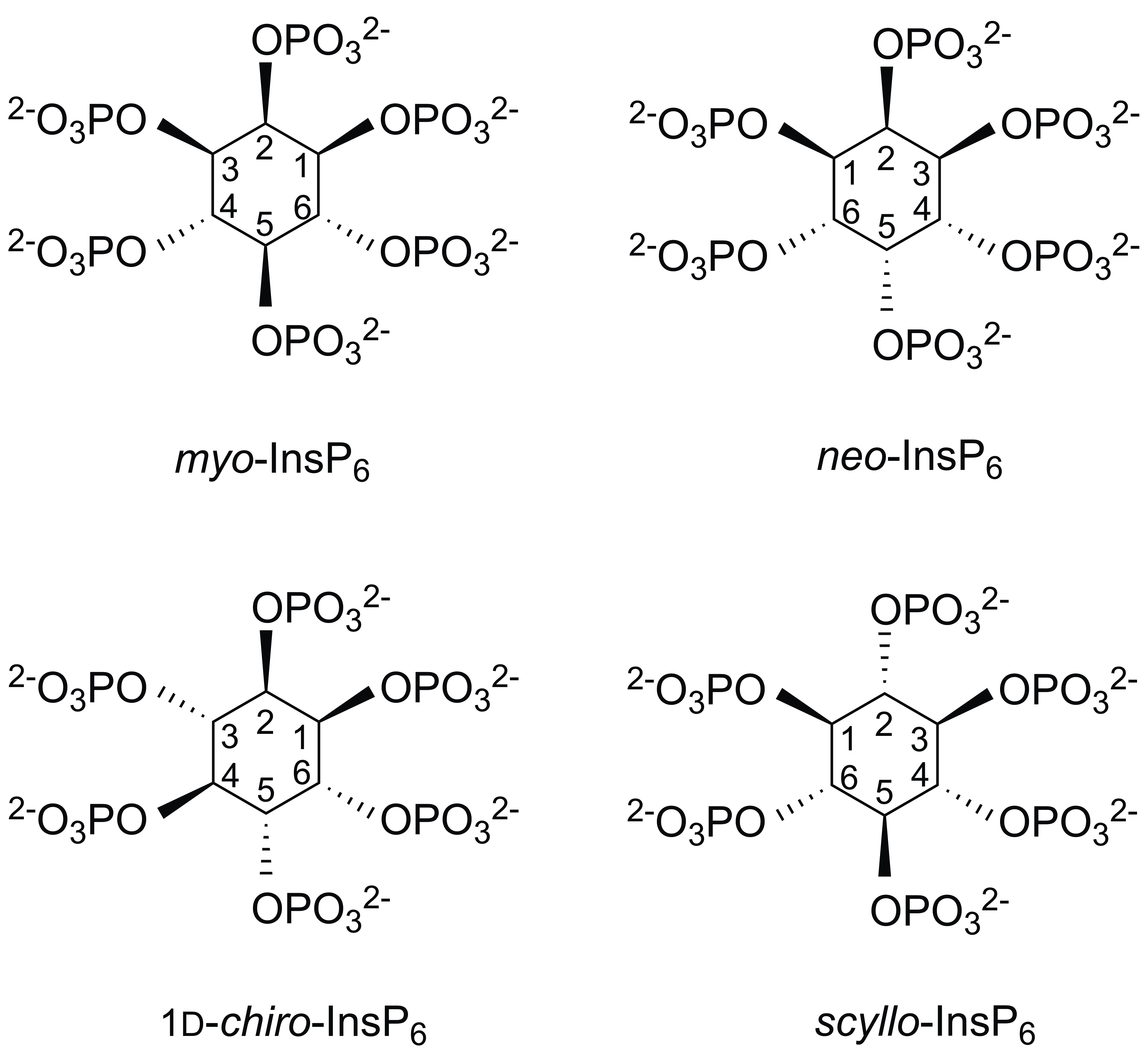
**

Figure S1. The structures of naturally occurring inositol hexakisphosphate displayed in the form of Mills projections.

In *myo*-InsP_6_, substituents on carbons 1-, 3-, 4-, 5- and 6- positions are in the equatorial position whilst that on carbon 2 is in the axial position. Carbons are numbered in *myo*-inositol according to the relaxation of IUPAC rules (IUB. 1989) that allow (for this isomer) use of the D-numbering convention.

IUB. 1989. Nomenclature Committee of the International Union of Biochemistry. Numbering of atoms in *myo*-inositol. *Biochem J* **258**: 1-2.

**
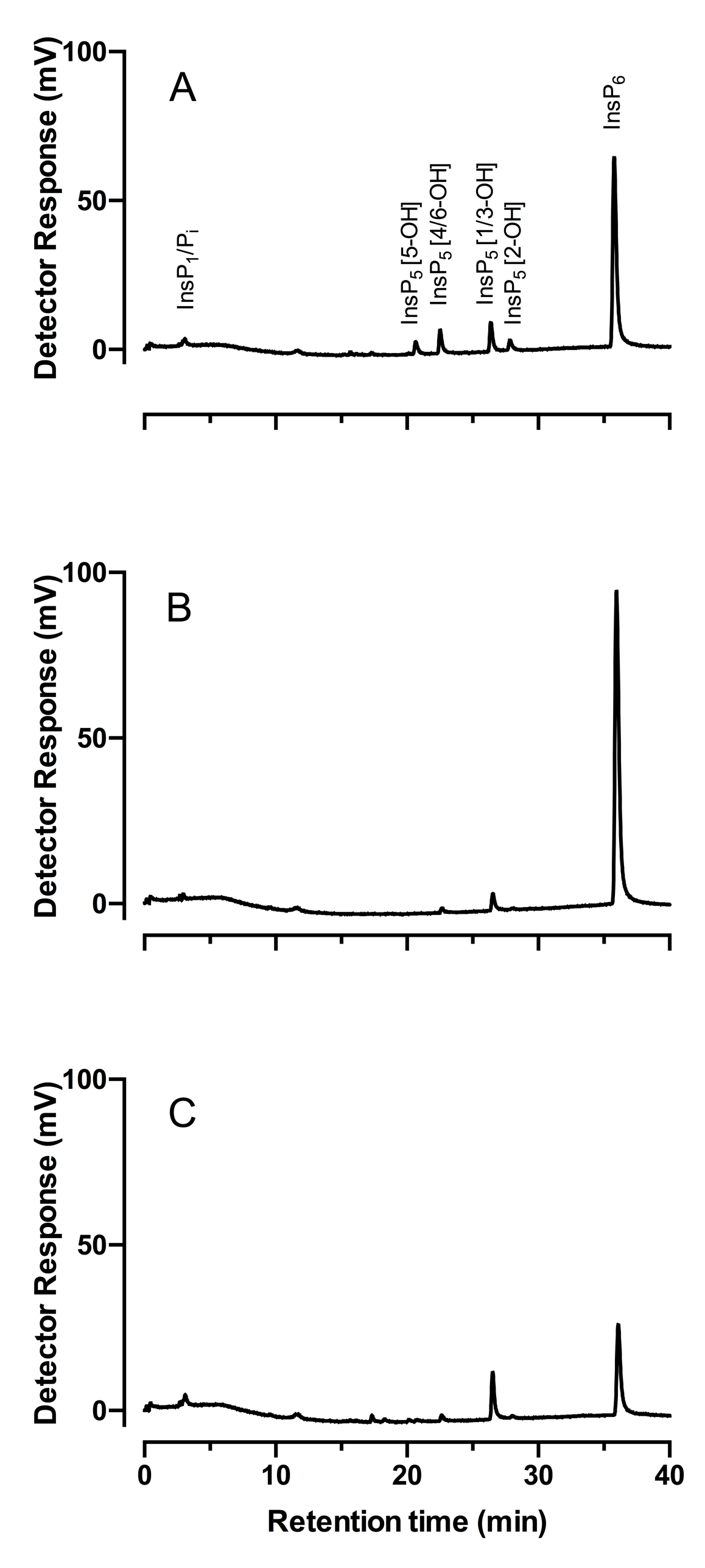
**

Figure S2. HPLC profiles of *as received* and autoclaved InsP_6_.

A, commercial InsP_6_ (Sigma P8810); B, a ‘clean’ InsP_6_ substrate; C, B after autoclaving at 120 °C, 7 kPa for 15 min. Significant impurities can confound phytase-discovery.


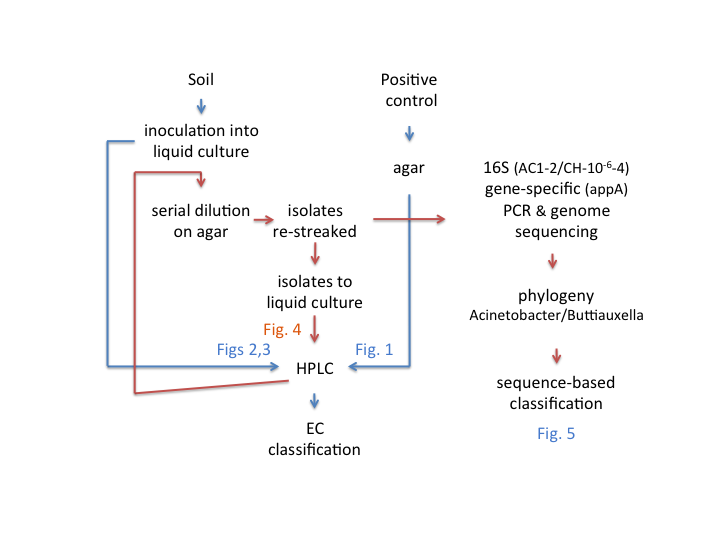


Figure S3. A schematic of workflows used.

EC, Enzyme Commission

Table S1 – Presence of canonical phytases among *Acinetobacter* and *Buttiauxella* spp. Genome Accession Numbers of a selection of thirty six *Acinetobacter* and nine *Buttiauxella* spp. representative of entries in Table 1: 1, AMSS01000011; 2, BBLI01000014; 3, CP020000; 4, JFYA01000008; 5, NGIR01000010; 6, KB849143; 7, RCWO01000001; 8, LQXZ01000001; 9, JEXG01000001; 10, BKXD01000021; 11, JABVBE010000148; 12, KB850261; 13, CP018259; 14, AP014630; 15, JICM01000056; 16, BKMB01000013; 17, UFMN01000002 (Glucose-1-phosphatase precursor); 18, FMYL01000002 (Histidine type phosphatase); 19, BKNN01000028; 20, POVU01000011; 21, KI530712 & KI530723; 22, FZLN01000003; 23, NEXW01000002; 24, RFFA01000043; 25, NOZT01000044; 26, KB849830; 27, VXKN01000015; 28, FMYL01000001; 29, JAACKC010000012; 30, LBNL01000031; 31, GG704964; 32, CP030880; 33, CP033768; 34, CP032134; 35, JAAZSP010000012; 36, QPHO01000725; 37, SNVY01000002; 38, BJFN01000002; 39, UIGI01000001; 40, LXEQ01000027; 41, JPRU01000002; 42, LXEP01000015; 43, LXER01000029; 44, QZWH01000027 (Bifunctional glucose-1-phosphatase/inositol phosphatase); 45, LXEO01000008 (Bifunctional glucose-1-phosphatase/inositol phosphatase). Genomes with a hit with an E value < 0.00005 are indicated, X.

|  |  | Phytase class | | | | |
| --- | --- | --- | --- | --- | --- | --- |
|  | Species or strain | Minpp | HAP | βPPhy | PTP | PAPhy |
| 1 | *Acinetobacter* sp. WC-141 | X | - | - | - | - |
| 2 | *Acinetobacter gerneri* | X | - | - | - | - |
| 3 | *Acinetobacter calcoaceticus* CA16 | X | - | - | - | - |
| 4 | *Acinetobacter baumannii* 573719 | X | - | - | - | - |
| 5 | *Acinetobacter pittii* ARLG1955 | X | - | - | - | - |
| 6 | *Acinetobacter calcoaceticus* NIPH 13 | X | - | - | - | - |
| 7 | *Acinetobacter calcoaceticus* RS3 | X | - | - | - | - |
| 8 | *Acinetobacter baumannii* MEX11594 | X | - | - | - | - |
| 9 | *Acinetobacte*r sp. 809848 | X | - | - | - | - |
| 10 | *Acinetobacter ursingii* TUM15527 | X | - | - | - | - |
| 11 | *Acinetobacter lactucae* KCJK9043 | X | - | - | - | - |
| 12 | *Acinetobacter ursingii* NIPH 706 | X | - | - | - | - |
| 13 | *Acinetobacter bereziniae* XH901 | X | - | - | - | - |
| 14 | *Acinetobacter guillouiae* NBRC 110550 | X | - | - | - | - |
| 15 | *Acinetobacter* sp. ETR1 | X | - | - | - | - |
| 16 | *Acinetobacter oleivorans* TUM15236 | X | - | - | - | - |
| 17 | *Acinetobacter baumannii* 4300STD | - | X | - | - | - |
| 18 | *Acinetobacter boissieri* ANC 4422 | - | X | - | - | - |
| 19 | *Acinetobacter brisouii* TUM15274 | - | X | - | - | - |
| 20 | *Acinetobacter* sp. MB5 | - | X | - | - | - |
| 21 | *Acinetobacter nectaris* CIP 110549 | - | X | X | - | - |
| 22 | *Acinetobacter apis* ANC 5114 | - | - | X | - | - |
| 23 | *Acinetobacter indicus* KCTC 42012 | - | - | X | - | - |
| 24 | *Acinetobacter ursingii* TG29426 | - | - | X | - | - |
| 25 | *Acinetobacter* sp. YT-02 | - | - | X | - | - |
| 26 | *Acinetobacter iwoffi* ATCC 9957 | - | - | X | - | - |
| 27 | *Acinetobacter qingfengsis* CCUG 69710T | - | - | X | - | - |
| 28 | *Acinetobacter boissieri* ANC 4422 | - | - | X | - | - |
| 29 | *Acinetobacter* sp. PS-1 | - | - | X | - | - |
| 30 | *Acinetobacter tandoii* SC36 | - | - | X | - | - |
| 31 | *Acinetobacter johnsonii* SH046 | - | - | X | - | - |
| 32 | *Acinetobacter haemolyticus* HW-2A | X | - | X | - | - |
| 33 | *Acinetobacter baumannii* FDAARGOS | X | - | X | - | - |
| 34 | *Acinetobacter chinensis* WCHAc010005 | - | - | X | - | - |
| 35 | *Acinetobacter cumulans* AC1 | - | - | X | - | - |
| 36 | *Acinetobacter baumannii* MH6 | - | - | - | - | X |
| 37 | *Buttiauxella* sp. JUb87 | - | X | - | - | - |
| 38 | *Buttiauxella* sp. A111 | - | X | - | - | - |
| 39 | *Buttiauxella agrestis* NCTC12119 | - | X | - | - | - |
| 40 | *Buttiauxella ferragutiae* ATCC 51602 | - | X | - | - | - |
| 41 | *Buttiauxella noackiae* MCE | - | X | - | - | - |
| 42 | *Buttiauxella gaviniae* ATCC 51604 | - | X | - | - | - |
| 43 | *Buttiauxella brennerae* ATCC 51605 | - | X | - | - | - |
| 44 | *Buttiauxella izardii* CCUG 35510 | - | X | - | - | - |
| 45 | *Buttiauxella noackiae* ATCC 51607 | - | X | - | - | - |
